# Steady-State and Time-Resolved Fluorescence Study of Selected Tryptophan-Containing Peptides in an AOT Reverse Micelle Environment

**DOI:** 10.3390/ijms242015438

**Published:** 2023-10-22

**Authors:** Krystian Gałęcki, Agnieszka Kowalska-Baron, Katarzyna E. Nowak, Anna Gajda, Beata Kolesińska

**Affiliations:** 1 Institute of Natural Products and Cosmetics, Faculty of Biotechnology and Food Sciences, Lodz University of Technology, Stefanowskiego St. 2/22, 90-537 Lodz, Poland; agnieszka.kowalska-baron@p.lodz.pl; 2 Department of Oncobiology and Epigenetics, Faculty of Biology and Environmental Protection, University of Lodz, Pomorska St. 141/143, 90-236 Lodz, Poland; katarzyna.nowak@biol.uni.lodz.pl; 3 Institute of Organic Chemistry, Faculty of Chemistry, Lodz University of Technology, Żeromskiego St. 114, 90-924 Lodz, Poland; anna.gajda@p.lodz.pl (A.G.); beata.kolesinska@p.lodz.pl (B.K.)

**Keywords:** tryptophan fluorescence, reverse micelles, fluorescence lifetime

## Abstract

The aim of this study was to demonstrate the utility of time-resolved fluorescence spectroscopy in the detection of subtle changes in the local microenvironment of a tryptophan chromophore in a confined and crowded medium of AOT reverse micelles, which mimic biological membranes and cell compartmentalization. For this purpose, fluorescence properties of L-tryptophan and several newly synthesized tryptophan-containing peptides in buffer and in an AOT reverse micelle medium were determined. It was shown that insertion of tryptophan and its short di- and tripeptides inside micelles led to evident changes in both the steady-state emission spectra and in fluorescence decay kinetics. The observed differences in spectral characteristics, such as a blue shift in the emission maxima, changes in the average fluorescence lifetime, and the appearance of environmental-dependent fluorescent species, showed the utility of time-resolved fluorescence spectroscopy as a sensitive tool for detecting subtle conformational modifications in tryptophan and its peptides induced by changes in polarity, viscosity, and specific interactions between chromophores and water molecules/polar groups/ions that occur inside reverse micelles.

## 1. Introduction

Many important biochemical reactions proceed within a confined aqueous microenvironment such as membranous organelles, cells, or endosomes. In such a microenvironment, there are high concentrations of substances dissolved in a crowded aqueous medium, which has properties that differ from those of bulk water.

Reverse micelles (RMs) can be considered a model system for a crowded microenvironment that contains a limited number of water molecules. Molecular organization in reverse micelles is similar to pockets of water in bioaggregates (such as biomembranes). Thus, RMs are excellent models for biological membranes and compartmentalization [1,2,3].

RMs are spherical self-aggregates of amphiphilic surfactants that contain nanometer-sized water droplets that are suspended in a bulk organic solvent. The polar headgroups of the surfactant are directed towards the water droplet, while the hydrophobic hydrocarbon chains orient towards the bulk organic solvent.

The critical parameter in the formation of RMs is water loading (*w*), which is defined as the molar ratio of water to surfactant [1]. Water loading directly affects the physical parameters of RM particles, including the size of the particle, the degree of solvation of a hosted molecule, and the encapsulation efficiency.

Crowded environments affect several important physical properties of proteins, including protein folding and stability, the effective volume of the folded protein, protein aggregation, and local protein dynamics. Moreover, under crowded conditions, such as those found within cells, translational diffusional rates are slowed by approximately one order of magnitude. Therefore, in RMs, the biological activity of enzymes may be modified [1,2,3].

The effects of confinement and crowding on protein dynamics inside RMs may arise from the following: (1) a reduction in the number of possible conformations in a polypeptide chain, which reduces the entropy of protein folding, and (2) the differences in the properties of bulk water due to specific interactions between water molecules and surrounding molecules of a surfactant, an ion, or a macromolecule, which are involved in the creation of a hydration shell [4,5].

RM-based studies are considered an excellent tool for examining the influence of in vivo-like conditions on the structure and dynamics of functional biomacromolecules. The application of RMs allows understanding the dynamics of solutes, especially polypeptides and proteins, located in the interfacial region [1,2,3].

The most frequently studied inverted micelles are those formed from the aerosol OT (di(2-ethylhexyl) sulfosuccinate) (AOT). Properties of the aqueous interior of AOT reverse micelles are different from those of bulk water, indicating strong interactions of water dipoles with sodium counterions and ionized sulfonate headgroups [6,7,8]. Moreover, so called “water pools” in the core of micelles differ from the layer of water molecules in close proximity to micellar vesicle walls.

The apparent dielectric constant of bound water inside AOT RMs is considerably lower than that of bulk water, which decreases its ability to solvate ions, and its viscosity is greatly increased. Sodium ions (Na^+^) may dissociate from AOT and become hydrated in the water core. The ratio of sodium ions to water may result in a micelle interior with high ionic strength. The nature of water in the core of inverted micelles of AOT in *n*-heptane is different from that of bulk water. This is caused by electrostatic ion-dipole attraction between sodium ions and water molecules. The Na^+^-water interaction energy is of the order of 25 kcal/mol [9]. Moreover, water molecules can interact with sulfonate groups of AOT. Polar headgroups of AOT may cause deprotonation of the carboxylic groups of amino acids. Hydration is the process that affects the stability and conformation of peptides in reverse micelles. The detergent and peptide polar groups have competing affinities for water molecules.

Conformational changes in polypeptides may be monitored by tryptophan fluorescence techniques since spectroscopic parameters of tryptophan fluorescence such as fluorescence quantum yield, fluorescence lifetime, and positions of fluorescence maxima are very sensitive to the subtle changes in the microenvironment of a tryptophan chromophore. Thus, steady-state and time-resolved spectroscopy may be applied to study conformational changes in tryptophan-containing polypeptides [10,11].

The photophysics of tryptophan (W) has been extensively studied by applying both steady-state and time-resolved fluorescence spectroscopy [12,13,14,15]. It was previously demonstrated that fluorescence decay of an aqueous solution of tryptophan is described by double-exponential kinetics. The two components, τ_1_ = 3.14 ns and τ_2_ = 0.51 ns (at pH = 7.0, 20 °C), have fluorescence emission maxima at 350 and 335 nm, respectively [12]. The relative proportions of the two components vary with emission wavelength. The origin of this behavior was assigned by Szabo and Rayner [12] to the presence of two excited 1La and 1Lb solvent relaxed states or to the existence of different rotamers or conformers of the alanyl side chain of tryptophan [15]. The fluorescence properties of tryptophan were also previously studied in micellar media. It was reported that tryptophan fluorescence decay in AOT/*n*-hexane reverse micelles is complex [16].

The fluorescence properties of short tryptophan-containing peptides have also been previously studied [14,17,18,19,20,21,22,23]. It is known that the fluorescence properties of tryptophan and tryptophan-containing peptides are affected by many factors, e.g., the temperature and pH of the environment [14,23]. Thus, p*K*a values of these compounds should be taken into consideration. It was previously reported that Trp at pH 7.0 is completely zwitterionic [14].

On the other hand, the physical properties of RM solutions are dependent on *w* ([H_2_O]/[surfactant] ratio) and change especially at a low *w* [24]. It is still unknown if the pH inside a micelle is the same as that in a buffer.

Chen and coworkers [19] reported three fluorescence lifetimes of Ala-Trp (AW) at pH 5.5: 0.44 ns (0.43), 1.28 ns (0.55), and 3.64 (0.02). The authors stated that since the p*K*a of WA is 7.32, at pH 7.0, the observed fluorescence originated from the presence of both the anionic and zwitterionic forms of this dipeptide. More recent studies of Albani [24] on tripeptides with a tryptophan residue in the second position (Ala-Trp-Ala, Arg-Trp-Lys, Arg-Trp-Pro, Leu-Trp-Leu, Phe-Trp-Ala, and Phe-Trp-Phe, pH 7.0) have shown biexponential fluorescence decay kinetics with the lifetimes originating from two substructures of tryptophan in the excited state.

Fluorescence decay kinetics of proteins with a single tryptophan residue have been shown to display triexponential behavior [24], in which the two shortest lifetimes originate from tryptophan substructures inherent to the tryptophan structure independently of the surrounding microenvironment. The third fluorescence lifetime is attributed to the specific interactions between tryptophan and neighboring amino acid residues [24]. In proteins containing more than one tryptophan residue, multiexponential kinetics are also observed, and the ratio of the two components is wavelength-dependent [10].

In this study, we applied steady-state and time-resolved fluorescence spectroscopy techniques to investigate the effect of the presence of a peptide bond(s) as well as the impact of the presence and position of an amino acid residue(s) (alanine (A), methionine (M), cysteine (C), or tryptophan (W)) on tryptophan fluorescence characteristics in model systems that mimic the cell interior. Aerosol OT (AOT) di(2-ethylhexyl) sulfosuccinate in *n*-heptane was used as an anionic surfactant in the synthesis of water-containing inverted micelles, in which tryptophan and selected newly synthesized tryptophan-containing peptides (Ala-Trp (AW), Ala-Trp-Ala (AWA), Trp-Met-Trp (WMW), Trp-Trp-Met (WWM), and Trp-Trp-Cys (WWC)) were encapsulated in an aqueous environment. The dimensions of reverse micelles depend on the water-loading ratio, defined as the molar ratio of water to surfactant suspended in the organic phase (*w* = [H_2_O]/[surfactant]). In this study, micelles of *w* = 4 and *w* = 10 were prepared. The fluorescence decay kinetics and emission spectra of tryptophan and its selected derivatives in buffer and in micelles were characterized. The observed differences in the spectral characteristics indicated that time-resolved fluorescence spectroscopy is a sensitive and useful tool for detecting subtle changes in the direct microenvironment of a tryptophan chromophore.

## 2. Results and Discussion

### 2.1. Fluorescence Properties of Tryptophan (W) in an Aqueous and AOT/n-Heptane Reverse Micelle Medium

**Figure 1:**
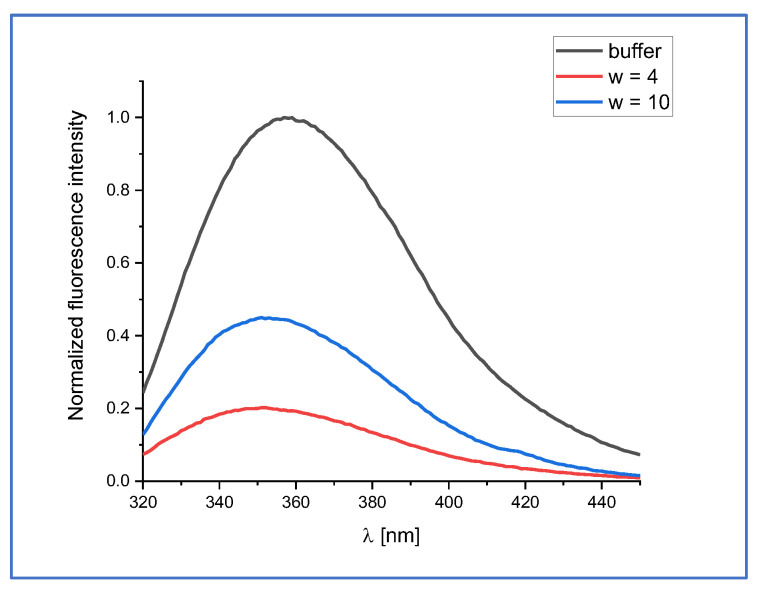
Normalized fluorescence emission spectra of tryptophan in buffer (pH 7.0) and in AOT/*n*-heptane micelles with different *w* values. The excitation wavelength was set to 295 nm.

The fluorescence spectra of aqueous tryptophan (phosphate buffer, pH 7.0) and in AOT micelles of different w are shown in Figure 1. It can be seen that the fluorescence emission maximum of tryptophan located inside the micelle is shifted towards shorter wavelengths. This blue shift is accompanied by a significant decrease in fluorescence intensity. Moreover, these changes in fluorescence parameters are more evident in micelles with a smaller *w*. This is the reason why only micelles with *w* = 4 were considered in the following part of this study.

The normalized steady-state fluorescence spectra of tryptophan in buffer and in micelles were fitted by applying Franck–Condon (FC) analysis of emission band profiles. The goodness of the fit was judged on the basis of detailed statistical analysis (see Supplementary Materials, Figures S1 and S2). The determined fitting parameters are presented in Table 1. The fluorescence of tryptophan arises from the presence of two conformers: one with a maximum located at around 340 nm and the second one at around 360 nm. Both these components are blue shifted when tryptophan is located inside the AOT micelles. Moreover, this blue shift is more evident in smaller micelles (*w* = 4) (15 nm and 7 nm at 360 nm and 340 nm, respectively). The relative contribution (see *f* in Table 1) of the shorter wavelength component is increased in the micelle environment, especially in smaller micelles (*w* = 4). It seems reasonable to explain the data in Figure 1 in terms of variations in the effective polarity and viscosity of the micellar interior with the size of water pockets. The blue shift may be associated with the decreased polarity of the micellar interior, changes in hydration of the chromophore inside the micelles, and with different properties of water in the local microenvironment of the indole chromophore.

The parameters of fluorescence decay kinetics of tryptophan in buffer and inside AOT micelles are presented in Table 2 (see Figure S3 for fluorescence kinetics curves).

As can be observed, the fluorescence decay kinetics of W in buffer is double-exponential with two components, namely, ~1 ns and ~3 ns, and the longer lifetime component is dominant (94%) when the emission wavelength of the decay is set to 360 nm. Therefore, the long lifetime component may be assigned to the longer wavelength component of the spectra (Table 1). In turn, the shorter lifetime component may be assigned to the shorter wavelength fluorescence band (~340 nm) obtained from FC analysis (Table 1), while the 3 ns component most probably has a fluorescence maximum at 360 nm (Table 1). This is in line with previous literature data in [12] and in [14], which reported that fluorescence decay kinetics of L-tryptophan in water (pH 7.0, λ*_ex_* = 296 nm, and λ*_em_* = 350 nm) are biexponential with the lifetimes 0.43 ns (4.71%) and 3.06 ns (95.29%). Small discrepancies in the determined fluorescence decay parameters (Table 2) may be due to different experimental conditions. The 3 ns and 1 ns components of the fluorescence decay kinetics of W correspond to various conformers resulting from different rotation around a C_α_–C_β_ bond. The different fluorescence lifetimes of the conformers arise from the different distances between the indole group and the acceptor group in the charge-transfer interactions [12,14].

In the AOT-micelle environment, the fluorescence decay kinetics of tryptophan are triexponential with ~0.5 ns, ~2 ns, and ~5 ns lifetime components. The ~0.5 ns and ~2 ns components may be assigned to the shorter and longer wavelength species of a tryptophan zwitterion, respectively. According to ref. [14], these two fluorescence lifetimes are inherent to the structure of tryptophan independently of the surrounding environment. The contribution of the ~1 ns fluorescence lifetime component is increased as compared to that in buffer. Additionally, in micelles, the relative contribution of the longer wavelength component of fluorescence lifetime (*τ*_2_) is decreased as compared to aqueous tryptophan, and this decrease is more evident in micelles with *w* = 4 (Table 2). The 5 ns component most probably may be attributed to the different tryptophan species that are immobilized/trapped inside the micelles and interact with the polar groups of AOT. In other words, the third lifetime component of fluorescence decay kinetics of tryptophan in micelles arises from specific interactions of tryptophan with polar groups of AOT and ions within the micelle interior, which may lead to the formation of a more rigid and compact structure of the tryptophan conformer. Thus, the third component reflects the local microenvironment of the fluorophore.

According to literature data, the p*K*_a_ of the carboxyl group of tryptophan is 2.83, the p*K*_a_ of the ammonium ion of tryptophan is 9.39 [25] or 9.5 ± 0.03 (pH 7.0, 23 °C) [14], and the pI of tryptophan is 5.89 [26]; therefore, at pH 7.0, zwitterionic species of tryptophan prevail, and interactions with polar groups of AOT inside the micelle environment are expected. The average fluorescence lifetime of aqueous tryptophan is decreased in the AOT-micelle environment (Table 2). This may be a result of more effective quenching interactions with ions present in the more crowded micelle interior. The fluorescence decay kinetics of tryptophan in AOT micelles showed the presence of three species (see Table 2), while FC analysis of the steady-state fluorescence spectra revealed the presence of only two fluorescent species (see Table 1). This may result from the limitation of the program used for FC analysis, which is not able to resolve two fluorophores with two closely aligned fluorescence maxima. Therefore, it may be suggested that the maximum fluorescence emission of the fluorophore population that corresponds to the third lifetime component overlaps with that of one of the shorter lifetime tryptophan conformers.

### 2.2. Influence of the Presence of a Peptide Bond(s) and an Alanine (A) Residue(s) on Tryptophan (W) Fluorescence Properties in an Aqueous and AOT/n-Heptane Reverse Micelle Medium

**Figure 2:**
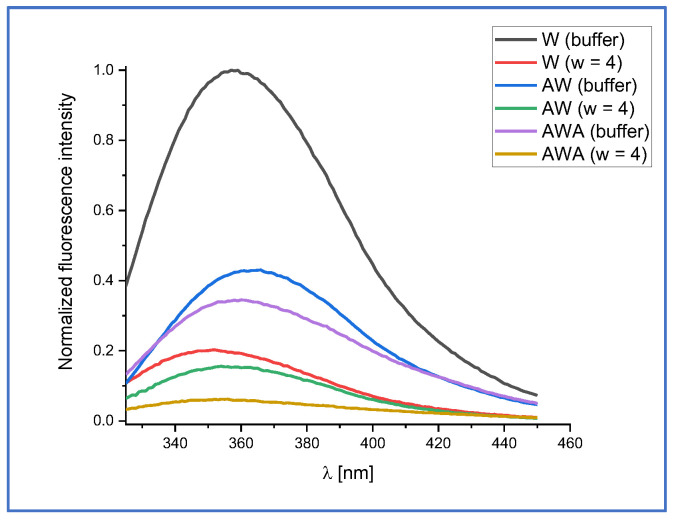
Normalized fluorescence emission spectra of W, AW, and AWA in buffer (pH 7.0) and in AOT/*n*-heptane micelles (*w* = 4). The excitation wavelength was set to 295 nm.

The fluorescence emission spectra of AW and AWA (and for comparison also aqueous W) in phosphate buffer (pH 7.0) and in AOT micelles (*w* = 4) are shown in Figure 2, from which it can be seen that the fluorescence intensity of AW is lower than that of W, and the fluorescence intensity of the former is higher than that of AWA. This may indicate that the presence of a peptide bond and an alanyl residue leads to the quenching of tryptophan fluorescence. Similarly as for W (Section 2.1), incorporation of AW and AWA inside the micelle leads to a blue shift and to a decrease in fluorescence intensity (Figure 2) as a result of a decrease in polarity of the micellar interior. Moreover, the alanyl residue is nonpolar, so the local environment around the indole ring of tryptophan is less polar in AW and AWA than in W.

The fitting parameters obtained from FC analysis of the steady-state fluorescence spectra of AW and AWA are gathered in Table 3. The parameters of the fluorescence decay kinetics of AW and AWA in buffer and inside AOT micelles are presented in Table 4 (see Figure S3 for fluorescence kinetics curves).

The fluorescence spectra of AW in buffer arise from two conformers with a maximum at about 360 nm and 340 nm, with a slight prevalence of the long wavelength component (Table 3). This is in line with the determined parameters of fluorescence decay kinetics (Table 4): the longer wavelength (~360 nm) component is characterized by the fluorescence lifetime of ~3 ns, while the shorter wavelength component (~340 nm) has a lifetime of 0.57 ns. The longer lifetime component prevails. To the best of our knowledge, there are only a few studies reporting the parameters of fluorescence decay kinetics of dipeptides with C-terminal tryptophan residues. According to ref. [17], the fluorescence decay kinetics of AW in aqueous solution (pH 7.0) at 18 ± 1 °C is biexponential with *τ*_av_ = 1.4 ± 5% ns (*τ*_1_ = 0.73 ± 5% ns and *τ*_2_ = 2.0 ± 5% ns). The fluorescence decay of GW at pH 7.0 was previously reported [14] to be biexponential with the lifetimes of 0.33 ns and 1.39 ns and the amplitudes 0.42 and 0.58, respectively. Interestingly, the fluorescence decay kinetics of WG, with an *N*-terminal tryptophan residue, at pH 7.0, was previously described [14] as triexponential with lifetimes of 0.8 ns (amplitude 0.16), 1.92 ns (amplitude 0.71), and 7.49 ns (amplitude 0.13) [14]. For AW, *τ*_av_ = 1.82 ns (Table 4) corresponds to the value from the literature of 1.4 ns [17] and is shorter than that for W (2.97 ns) (Table 2). It may be related to the presence of a peptide bond and C-terminal W in AW. Chang and coworkers [14] reported on static self-quenching in X-Trp dipeptides.

In micelles, the fluorescence decay kinetics of AW is triexponential with lifetimes of 1.17 ns (37%), 2.91 ns (39%), and 6.55 ns (24%) (Table 4). The first two fluorescence decay components arise from tryptophan, while the longest lifetime, most probably, is related to a different conformer of AW, which is generated by specific environmental conditions inside the micelles (lower polarity, confined space) and by specific interactions of AW with polar groups of AOT, molecules of water, and ions.

FC analysis of the fluorescence spectra of AW in micelles, AWA, and other tripeptides both in buffer and in micellar medium showed only one component of the spectra (see Table 3 and Table 5), although fluorescence decay of these peptides is multiexponential (see Table 4 and Table 6). This probably is due to the low fluorescence signal of the AW and AWA samples.

The determined parameters of fluorescence decay kinetics of AWA in buffer (Table 4) indicated biexponential behavior with lifetimes of ~1 ns and ~3 ns and predominance of the shorter component, while in AOT micelles, the decay of AWA becomes triexponential with lifetimes of 0.79 ns, 3.46 ns, and 6.83 ns, but the ~3 ns component prevails. According to the literature [24], the fluorescence lifetimes (measured at λ*_em_* = 350 nm) of AWA in pure distilled water are 0.62 ns and 1.76 ns, but the contributions of these components were not given.

Moreover, the average fluorescence lifetime of AW and AWA in micelles, as compared to that in buffer, is increased (Table 4). This result is in contrast to that for tryptophan, which showed, probably due to more effective quenching inside micelles, a decrease in the fluorescence lifetime in micelles (Table 2). It may be postulated/hypothesized that the observed increased fluorescence lifetime of AW and AWA in AOT micelles is related to a different, more compact conformer of the peptides in which the tryptophan residue is more shielded and is not exposed to the quenching interactions with surrounding water molecules, AOT polar groups, and ions. This fluorescent species/conformer is the result of conformational dynamics of the peptide inside micelles and gives rise to the observed triexponential fluorescence decay of the ~7 ns component. According to literature data [24], the fluorescence decay kinetics of single tryptophan containing proteins are triexponential, where the two shortest lifetime components are assigned to the tryptophan substructures formed in the excited state, while the third ((~4–9 ns) depending on the protein) component arises from specific interactions of tryptophan with neighboring amino acid residues.

### 2.3. Influence of the Presence and Position of the Amino Acid Residue on Tryptophan (W) Fluorescence Properties in Aqueous and AOT/n-Heptane Reverse Micelle Medium

**Figure 3:**
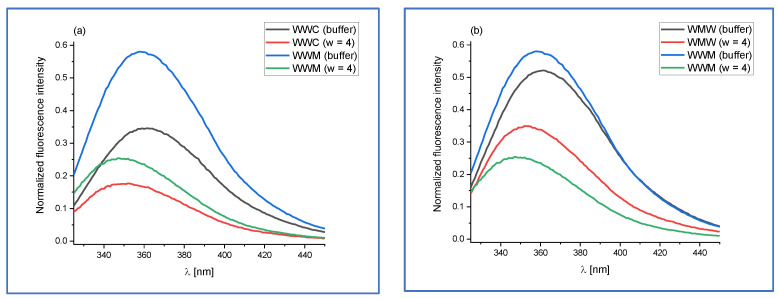
Normalized fluorescence emission spectra of (**a**) WWC and WWM and (**b**) WMW and WWM in buffer (pH 7.0) and AOT/*n*-heptane micelles (*w* = 4). The excitation wavelength was set to 295 nm. The values of fluorescence intensities of peptides were normalized to those for tryptophan in buffer.

The normalized fluorescence spectra of WWC and WWM in buffer and in AOT micelles are compiled in Figure 3a, while the spectra of WMW and WWM in buffer and in AOT micelles are presented in Figure 3b.

From Figure 3, it may be noticed that the fluorescence emission maxima of WWM, WWC, and WMW are blue shifted in micellar media as compared to those in buffer. The blue shift is accompanied by a decrease in fluorescence intensity, which is more evident in Figure 3a than in Figure 3b. It may be suggested that the change in the position of the amino acid residue in the tripeptide chain affects fluorescence spectra to a lesser degree than the change in the amino acid type (C to M).

The results of FC analysis of the fluorescence spectra of WWC, WWM, and WMW in buffer and AOT micelles are gathered in Table 5, which, in contrast to the determined fluorescence decay kinetics parameters (see Table 6), revealed only one fluorescent component in the spectra. This is surprising since the emission spectra, especially for WMW in buffer (Figure 3), clearly show the presence of at least two species, as a shoulder can be seen at the red edge. This may arise from the limitation of the program used to fit the data to the FC model.

However, the fluorescence maximum corresponding to this component is blue shifted in micellar media in comparison with buffer (16 nm, 12 nm, and 9 nm for WWC, WWM, and WMW, respectively). The fluorescence spectra for WWM (both in buffer and micelles) is slightly more blue shifted as compared to those for WWC, which may be explained by the difference in the polarity of these C-terminal amino acids (methionine is less polar as compared to cysteine).

The parameters of the fluorescence decay kinetics of WWC, WWM, and WMW in buffer and inside AOT micelles are presented in Table 6 (see Figure S3 for fluorescence kinetics curves).

The fluorescence decay kinetics of the studied tripeptides in buffer are biexponential with the lifetimes of ~1 ns and ~3 ns, which can be assigned to different tryptophan conformers. For WWC and WWM, the predominance of the longer lifetime component may be observed, while for WMW, the shorter lifetime component slightly prevails, which indicates that fluorescence decay kinetics parameters may detect the position of amino acid residue X in WWX and WXW tripeptides. In AOT micelles, the triexponential decay kinetics of the tripeptides containing two tryptophan residues were observed, where the two shortest lifetime components may be assigned to tryptophan residues. What is interesting is that in the micellar medium, these two short lifetime components of the decay are shorter for WWC and WWM as compared to those for WMW, which indicates a different mechanism of excited-state deactivation in WWX tripeptides. Moreover, the contribution of the third, the longest lifetime component for WMW, in micelles is smaller as compared to those for WWC and WWM. These changes in relative contributions of the fluorescence decay components indicate that time-resolved fluorescence spectroscopy is able to detect subtle changes in both polarity and location of amino acid residues in tryptophan-containing peptides located inside reverse micelles.

In buffer, the average fluorescence lifetimes τ*_av_* of all the studied peptides (see Table 4 and Table 6) are lower as compared to that of tryptophan (Table 2), which may be caused by quenching by charge transfer from the indole ring to a peptide bond(s). In micellar medium, τ_av_ of WMW as well as of AW and AWA is increased as compared to that in buffer. On the other hand, τ*_av_* of W, WWC, and WWM are shorter as compared to those in buffer. It should also be noted that for peptides in which W is at the C-terminal position, τ_av_ in solution is shorter (AW 1.82 ns (Table 4) and WMW 1.86 ns (Table 6)) and in micelles is longer (AW 3.15 ns (Table 4) and WMW 2.43 ns (Table 6)) than that for tryptophan. The tryptophan fluorescence in those peptides in solution most probably is quenched by a peptide bond(s) as a result of charge transfer from the indole ring to the electrophilic carbonyl group. It may be suggested that this mechanism of quenching does not occur in AOT micelles. The observed differences in spectral characteristics in peptides containing two neighboring tryptophan residues (WWC and WWM) suggest their more complex photophysics, most probably due to the presence of specific tryptophan-tryptophan interactions.

## 3. Materials and Methods

### 3.1. Materials

L-tryptophan (W), *n*-heptane, and Aerosol OT (AOT) di(2-ethylhexyl)sulfosuccinate were purchased from Sigma-Aldrich (St. Louis, MO, USA) and used without further purification. *n*-heptane was dehydrated. High-purity (Millipore, Merck, Darmstadt, Germany) doubly distilled water (18.2 MΩ at 25 °C) was used for all the experiments. Peptides (AW, AWA, WMW, WWM, and WWC) were synthesized as described in [27].

### 3.2. Methods

#### 3.2.1. Preparation of Water-Containing AOT Inverted Micelles

A 0.1 M AOT solution in *n*-heptane was mixed (with the use of a magnetic stirrer) with a 10^−5^ M solution of tryptophan and selected peptides (AW, AWA, WMW, WWM, and WWC) in 0.005 M phosphate buffer (pH 7.0). The ratio of volumes of the abovementioned solutions corresponded to *w* = 4 and *w* = 10.

#### 3.2.2. Steady-State Fluorescence Measurements

Steady-state fluorescence measurements were performed with a FluoroMax 4 (Jobin Yvon SPEX Instruments S.A Inc., Warsaw, Poland) spectrofluorometer, using an excitation wavelength of 295 nm. All measurements were performed in a standard quartz cuvette at 25 °C.

#### 3.2.3. Franck–Condon (FC) Analysis of the Steady-State Fluorescence Spectra

The steady-state fluorescence emission spectra were normalized and fit by application of Franck–Condon (FC) analysis of emission band profiles, as described previously [28,29,30] using the following equations:

(1) $${I\left(\bar{\upsilon }\right)}_{i}=\sum_{\upsilon =0}^{4}\left\{{\left(\frac{E_{0}-\upsilon \hbar \omega }{E_{0}}\right)}^{3}\left(\frac{S^{\upsilon }}{\upsilon !}\right)exp\left[-4ln\left(2\right){\left(\frac{\bar{\upsilon }-E_{0}+\upsilon \hbar \omega }{{\Delta \bar{\upsilon }}_{1/2}}\right)}^{2}\right]\right\}$$

(2) $$I\left(\bar{\upsilon }\right)=f\cdot {I\left(\bar{\upsilon }\right)}_{1}+\left(1-f\right){I\left(\bar{\upsilon }\right)}_{2}$$

where ${I\left(\bar{\upsilon }\right)}_{i}$ is the emission intensity at the energy $\bar{\upsilon }$ in wavenumbers [cm^−1^] of the *i*-th component, relative to the intensity of the 0→0 transition, E_0_ is the energy gap between the zero vibrational levels of the ground and excited states, ℏω and S are the quantum spacing and the Huang–Rhys factor [31] reflecting the degree of distortion in the single, average mode as the difference in equilibrium displacements, ${\Delta \bar{\upsilon }}_{1/2}$ is the full width at half maximum for individual vibronic lines [30], and f is the fractional contribution of the ${I\left(\bar{\upsilon }\right)}_{i}$. The results of the fits were identical when the summation was carried out over four and six ground-state vibrational levels (υ = 0→4, υ = 0→6).

The parameters in the FC analysis were as follows: the 0-0 energy gap (E_0_); the bandwidth at half height (${\Delta \bar{\upsilon }}_{1/2}$), which includes the classical reorganization energy; the quantum spacing for the averaged acceptor mode (ℏω), and the electron-vibrational coupling constant or Huang–Rhys factor (S) [31]. Emission intensities were fit by optimizing the parameters E_0_, ${\Delta \bar{\upsilon }}_{1/2}$, ℏω, and S with a least squares minimization routine.

#### 3.2.4. Time-Resolved Fluorescence Measurements

Fluorescence lifetime measurements were carried out at 25 °C with a FL900CDT time-correlated single-photon counting fluorimeter from Edinburgh Analytical Instruments (Edinburgh, Scotland, UK) with a 295 nm excitation source (NanoLED-295 diode) having a pulse FWHM of ~1.18 ns. Data acquisition and analysis were performed using the software (F900, version 7.2.2) provided by Edinburgh Analytical Instrumentation (Edinburgh, Scotland, UK). Fluorescence decay kinetics were fitted using reconvolution fit analysis. The instrumental response function was determined using LUDOX solution (Sigma-Aldrich, St. Louis, MO, USA). The goodness of fit was estimated by using reduced χ^2^ values.

## 4. Conclusions

In this work, it was shown that the fluorescence behavior of the studied tryptophan-containing short peptides in a neutral aqueous solution is complex and changes significantly in small (*w* = 4) AOT/*n*-heptane RMs.

The observed changes in fluorescence characteristics indicated that insertion of tryptophan and its studied peptides into RMs led to a blue shift in the emission spectra and to a decrease in fluorescence intensity.

In buffer, the fluorescence decay kinetics of tryptophan and its peptides was biexponential, while in micelles, a third fluorescence lifetime component appeared. The two short lifetime components may be assigned to two different conformers of tryptophan, while the third component was attributed to a more rigid fluorophore structure, which is the result of specific interactions of tryptophan or its peptides with surrounding water molecules, ions, and polar groups of AOT.

By comparing the average fluorescence lifetime of tryptophan in buffer with that in micelles, it was noted that this parameter was decreased in the micellar medium in the case of tryptophan, WWC, and WWM, while insertion of AW, AWA, and WMW inside RMs led to an increase in the average fluorescence lifetime. It was postulated that in the micellar medium, there were more effective quenching interactions due to a more crowded environment. It may also be suggested that a confined micellar medium induced conformational changes in AW, AWA, and WMW, in which the tryptophan residue was shielded from collisional quenching interactions and the local environment of the chromophore was less polar, which in turn resulted in the increased average fluorescence lifetime.

The results of this study provide useful information that may be helpful for under-standing conformational dynamics in peptides, polypeptides, and proteins in model biological systems. Additionally, the obtained results show the utility of time-resolved fluorescence spectroscopy as a sensitive tool for detecting subtle conformational modifications in tryptophan and its peptides that occur inside reverse micelles.

## Figures and Tables

**Table 1:** The fitting parameters obtained from FC analysis of the fluorescence spectra of tryptophan in buffer (pH 7.0) and in AOT/*n*-heptane micelles.

	*f*	E_0_ [cm^−1^]	E_0_ [nm]	ℏω [cm^−1^]	S	${\Delta \bar{\upsilon }}_{1/2}$ [cm^−1^]
buffer	0.34	27,909	358	1508	0.71	2427
	0.66	29,366	340	2943	0.86	3209
*w* = 4	0.14	29,176	343	840	1.30	2023
	0.86	30,054	333	3234	1.29	3714
*w* = 10	0.30	28,031	357	85	0.53	2807
	0.70	29,689	337	3413	1.10	3159

**Table 2:** The parameters of the fluorescence decay kinetics (fluorescence lifetime components {τ_i_} and their fractional contributions {f_i_} (*i* = 1, 2 or *i* = 1, 2, 3)) of tryptophan in buffer (pH 7.0) and inside AOT/*n*-heptane micelles.

	τ_1_ [ns] (f_1_ [%])	τ_2_ [ns] (f_2_ [%])	τ_3_ [ns] (f_3_ [%])	τ_av_^1^ [ns]
buffer	1.17 (6)	3.09 (94)	-	2.97
*w* = 4	0.57 (23)	2.17 (42)	5.20 (35)	2.85
*w* = 10	0.54 (26)	1.96 (57)	5.91 (17)	2.29

^1^ defined as $\tau_{av}=\sum f_{i}\cdot \tau_{i}$ (*i* = 1, 2 or *i* = 1, 2, 3); error <2%. Instrument parameters: counts = 10,000, slit = 10 nm, λ_ex_ = 295 nm, λ_em_ = 360 nm, and FWHM ~1.18 ns.

**Table 3:** The fitting parameters obtained from FC analysis of the fluorescence spectra of AW and AWA in buffer (pH 7.0) and in AOT/*n*-heptane micelles.

		*f*	E_0_ [cm^−1^]	E_0_ [nm]	ℏω [cm^−1^]	S	${\Delta \bar{\upsilon }}_{1/2}$ [cm^−1^]
buffer	AW	0.57	27,944	358	1975	0.85	2655
		0.43	29,516	339	2910	0.94	2917
	AWA	1.00	28,257	354	3420	0.55	5071
*w* = 4	AW	1.00	28,282	354	2742	0.26	4937
	AWA	1.00	28,685	349	3878	0.63	5063

**Table 4:** The parameters of the fluorescence decay kinetics (fluorescence lifetime components {τ_i_} and their fractional contributions {f_i_} (*i* = 1, 2 or *i* = 1, 2, 3)) of AW and AWA in buffer (pH 7.0) and inside AOT/*n*-heptane micelles.

		*τ*_1_ [ns] (f_1_ [%])	τ_2_ [ns] (f_2_ [%])	τ_3_ [ns] (f_3_ [%])	τ_av_^1^ [ns]
buffer	AW	0.57 (35)	2.48 (65)	-	1.82
	AWA	1.10 (68)	3.02 (32)	-	1.71
*w* = 4	AW	1.17 (37)	2.91 (39)	6.55 (24)	3.15
	AWA	0.79 (20)	3.46 (57)	6.83 (23)	3.72

^1^ defined as $\tau_{av}=\sum f_{i}\cdot \tau_{i}$ (*i* = 1,2 or *i* = 1, 2, 3); error < 2%. Instrument parameters: counts = 10,000, slit = 10 nm, λ*_ex_* = 295 nm, λ*_em_* = 360 nm, and FWHM ~1.18 ns.

**Table 5:** The fitting parameters obtained from FC analysis of the fluorescence spectra of WWC, WWM, and WMW in buffer (pH 7.0) and in AOT/*n*-heptane micelles.

		E_0_ [cm^−1^]	E_0_ [nm]	ℏω [cm^−1^]	S	${\Delta \bar{\upsilon }}_{1/2}$ [cm^−1^]
buffer	WWC	28,123	356	2252	0.39	5212
	WWM	28,481	351	2293	0.54	5294
	WMW	28,061	356	2668	0.37	4939
*w* = 4	WWC	29,407	340	2571	0.86	4171
	WWM	29,506	339	2639	0.78	4521
	WMW	28,848	347	2740	0.64	4194

**Table 6:** The parameters of the fluorescence decay kinetics (fluorescence lifetime components {τ*_i_*} and their fractional contributions {f*_i_*} (*I* = 1, 2 or *i* = 1, 2, 3) of WWC, WWM, and WMW in buffer (pH 7.0) and inside AOT/*n*-heptane micelles.

		τ_1_ [ns] (f_1_ [%])	τ_2_ [ns] (f_2_ [%])	τ_3_ [ns] (f_3_ [%])	τ_av_^1^ [ns]
buffer	WWC	1.06 (45)	3.77 (55)	-	2.55
	WWM	0.99 (32)	2.99 (68)	-	2.35
	WMW	1.08 (53)	2.74 (47)	-	1.86
*w* = 4	WWC	0.26 (27)	1.23 (42)	5.01 (31)	2.15
	WWM	0.56 (46)	1.83 (33)	5.50 (21)	2.02
	WMW	0.90 (42)	3.21 (53)	6.53 (5)	2.43

^1^ defined as $\tau_{av}=\sum f_{i}\cdot \tau_{i}$ (*i* = 1,2 or *i* = 1, 2, 3); error <2%. Instrument parameters: counts = 10,000, slit = 10 nm, λ*_ex_* = 295 nm, λ*_em_* = 360 nm, and FWHM ~1.18 ns.

## Data Availability

The data presented in this study are available in the article, and raw data are available upon request.

## Supplementary Materials

The following supporting information can be downloaded at https://www.mdpi.com/article/10.3390/ijms242015438/s1.

## Abbreviations

A	Amino acid Ala
AOT	Aerosol OT di(2-ethylhexyl) sulfosuccinate
AW	Dipeptide Ala-Trp
AWA	Tripeptide Ala-Trp-Ala
C	Amino acid Cys
FC	Franck–Condon
M	Amino acid Met
RMs	Reverse micelles
W	Amino acid Trp
WMW	Tripeptide Trp-Met-Trp
WWC	Tripeptide Trp-Trp-Cys
WWM	Tripeptide Trp-Trp-Met
