# Supplementary material for: Steady-State and Time-Resolved Fluorescence Study of Selected Tryptophan-Containing Peptides in an AOT Reverse Micelle Environment
Source: Int J Mol Sci. 2023 Oct 22;24(20):15438. doi: 10.3390/ijms242015438 (PMC10607525; doi:10.3390/ijms242015438)
Supplement: Supplementary file 1 [file ijms-24-15438-s001.zip › ijms-2656676-supplementary.pdf]

Steady-state and time-resolved fluorescence study on selected tryptophan containing peptides in AOT-reverse micelles environment

Krystian Gałęcki [1*], Agnieszka Kowalska-Baron [1], Katarzyna E. Nowak [2], Anna Gajda [3] and Beata Kolesińska [3]

1 Institute of Natural Products and Cosmetics, Faculty of Biotechnology and Food Sciences, Lodz University of Technology, Stefanowskiego St. 2/22, 90-537 Lodz, Poland; agnieszka.kowalska-baron@p.lodz.pl

2 Department of Oncobiology and Epigenetics, Faculty of Biology and Environmental Protection, University of Lodz, Pomorska St. 141/143, 90-236 Lodz, Poland; katarzyna.nowak@biol.uni.lodz.pl

3 Institute of Organic Chemistry, Faculty of Chemistry, Lodz University of Technology, Żeromskiego St. 114, 90-924 Lodz, Poland; beata.kolesinska@p.lodz.pl

- Correspondence:krystian.galecki@p.lodz.pl

Supplementary Materials

S1. Fluorescence emission spectral fitting (Franck-Condon (FC) analysis)

a) code to Origin Program of one component FC analysis:

```
y = y0 + exp(-2.7726*((x-a0)/a3)^2)+((a0-a1)/a0)^3*(a2)*(exp(-2.7726*((x-a0+a1)/a3)^2))+((a0-2*a1)/a0)^3*((a2^2)/2)*(exp(-2.7726*((x-a0+(2*a1))/a3)^2))+((a0-3*a1)/a0)^3*((a2^3)/6)*(exp(-2.7726*((x-a0+(3*a1))/a3)^2))+((a0-4*a1)/a0)^3*((a2^4)/24)*(exp(-2.7726*((x-a0+(4*a1))/a3)^2))
```

where: y = normalized fluorescence intensity, x – wavenumber [cm^{-1}], a0 – E_0 [cm^{-1}], a1 - $\hbar\omega$ [cm^{-1}], a2 – S, a3 - $\Delta\bar{\upsilon}_{1/2}$ [cm^{-1}]

b) code to Origin Program of two component FC analysis:

```
y = y0+a*(exp(-2.7726*((x-a0)/a3)^2)+((a0-a1)/a0)^3*(a2)*(exp(-2.7726*((x-a0+a1)/a3)^2))+((a0-2*a1)/a0)^3*((a2^2)/2)*(exp(-2.7726*((x-a0+(2*a1))/a3)^2))+((a0-3*a1)/a0)^3*((a2^3)/6)*(exp(-2.7726*((x-a0+(3*a1))/a3)^2))+((a0-4*a1)/a0)^3*((a2^4)/24)*(exp(-2.7726*((x-a0+(4*a1))/a3)^2)))+(1-a)*(exp(-2.7726*((x-a4)/a7)^2)+((a4-a5)/a4)^3*(a6)*(exp(-2.7726*((x-a4+a5)/a7)^2))+((a4-2*a5)/a4)^3*((a6^2)/2)*(exp(-2.7726*((x-a4+(2*a5))/a7)^2))+((a4-3*a5)/a4)^3*((a6^3)/6)*(exp(-2.7726*((x-a4+(3*a5))/a7)^2))+((a4-4*a5)/a4)^3*((a6^4)/24)*(exp(-2.7726*((x-a4+(4*a5))/a7)^2)))
```

where: y = normalized fluorescence intensity, x – wavenumber [cm^{-1}], a – the fractional contribution, a0 and a4 – E_0 [cm^{-1}], a1 and a5 - $\hbar\omega$ [cm^{-1}], a2 and a6 – S, a3 and a7 - $\Delta\bar{\upsilon}_{1/2}$ [cm^{-1}], a0,a1,a2,a3 are the parameters for the first component, a4,a5,a6,a7 for the second component.

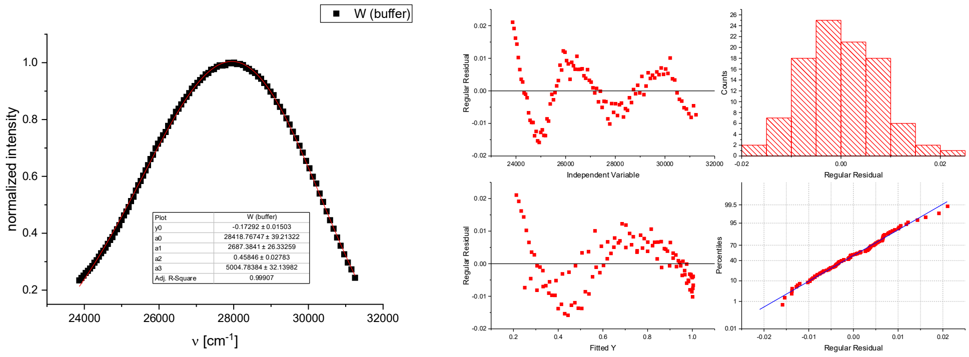

Figure S1. The fluorescence spectra fitting FC function (left) of one component and the statistical analysis of fit (right) for the tryptophan in buffer (pH 7.0).

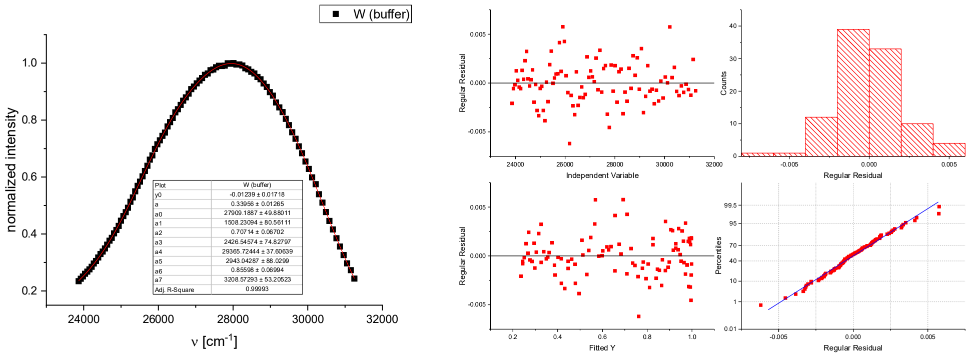

Figure S2. The fluorescence spectra fitting FC function (left) of two components and the statistical analysis of fit (right) for the tryptophan in buffer (pH 7.0).

S2. Fluorescence kinetics curves

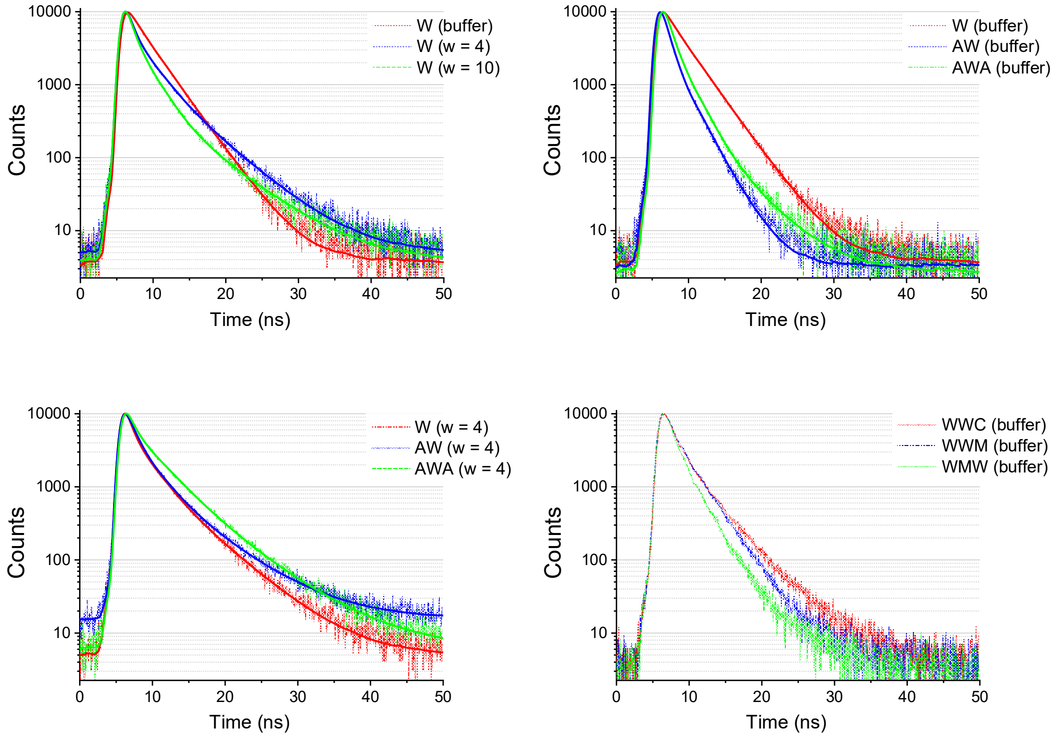

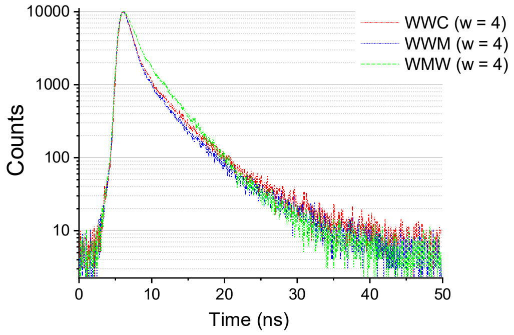

Figure S3. The fluorescence intensity decays and fitting curves for multi-exponent decay model for tryptophan (W) and the studied peptides (AW, AWA, WWC, WWM and WMW) in buffer (pH 7.0) and AOT/*n*-heptane-reverse micelles (*w*=4). Excitation wavelength was 295 nm and emission wavelength was 360 nm.
